# A complex case of bacterial pericarditis caused by a new pathogenic agent

**DOI:** 10.3205/id000088

**Published:** 2024-09-11

**Authors:** Joana Lima Lopes, Daniel Candeias Faria, Bárbara Flor-de-Lima, Márcio Madeira, Sara Ranchordas, José Pedro Neves, João Baltazar Ferreira

**Affiliations:** 1 Cardiology Department, Hospital Professor Doutor Fernando Fonseca, Amadora, Portugal; 2 Infectious Diseases Department, Hospital Professor Doutor Fernando Fonseca, Amadora, Portugal; 3 Cardiac Surgery Department, Hospital de Santa Cruz, Carnaxide, Portugal

**Keywords:** infectious pericarditis, Dermabacter hominis, aggressive agent, pericardiectomy

## Abstract

*Dermabacter hominis* is a gram-positive facultative anaerobic agent. It is a human skin colonizer that can be responsible for opportunistic infections in immunocompromised patients. To date, the infections caused by this agent are related to bone, joint, eye, peritoneal dialysis catheters, abscesses or infected vascular grafts. Overall, it has a favorable outcome with good response to vancomycin, teicoplanin or linezolide, and so it has not been considered a concerning pathogenic agent. We present the first case in scientific literature with isolation of *D. hominis* in pericardial fluid in the setting of infectious bacterial pericarditis, with an aggressive course and poor evolution.

## Introduction

*Dermabacter hominis* is a gram-positive facultative anaerobic agent seldom associated with human infections [1]. When infectious, it is usually in the setting of immunocompromised patients with comorbidities such as diabetes [2]. The few cases of human infections cause by *D. hominis* report its isolation in joint fluid, blood cultures, wound exudates, bone samples, skin and soft skin infections [3]. It is considered an opportunistic agent, with favorable outcome. We present a case reflecting opposite findings regarding its pathogenicity. Our case regards an aggressive bacterial pericarditis, with isolation of *D. hominis* in pericardial fluid, in a patient with persistently negative blood cultures. The case had a poor response to dual antibiotherapy and required cardiac surgery intervention with pericardiectomy. 

## Case description

**Figure 1:**
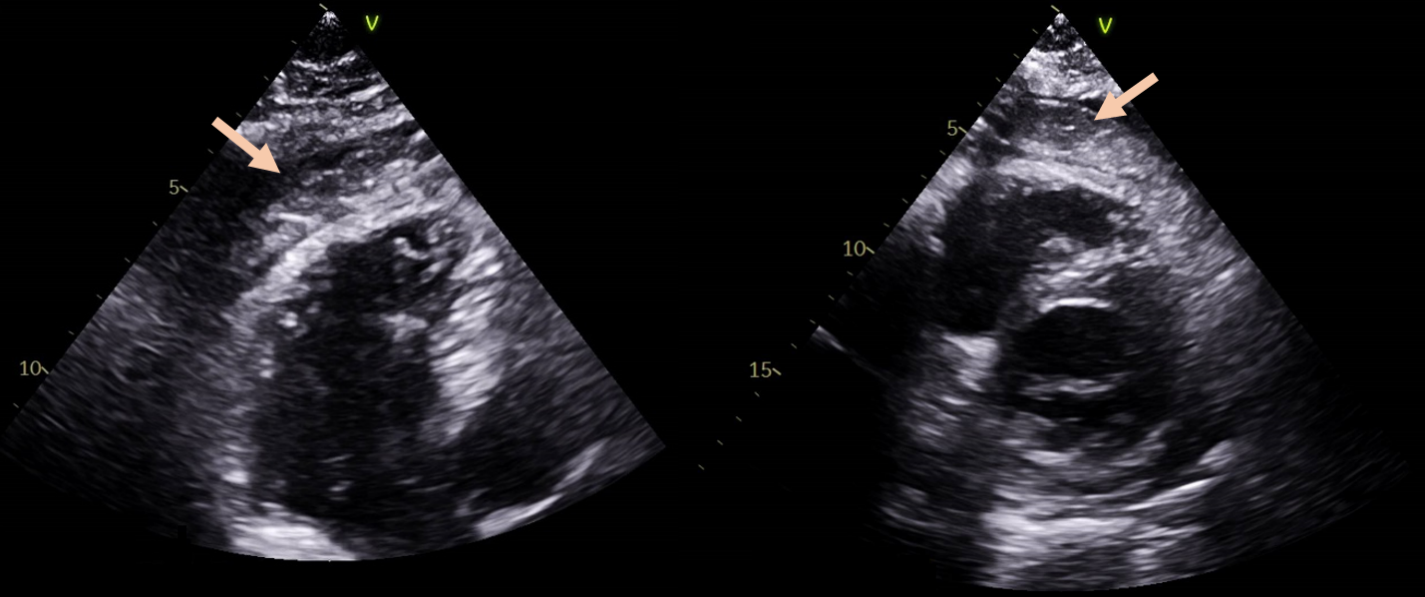
Re-development of pericardial effusion post-pericardiocentesis On the left: TTE image in 3 chamber view showing pericardial effusion with heterogeneous content in the pericardial space (arrow), inexistent in the TTE post pericardiocentesis; on the right: TTE image in short axis view, showing the same heterogeneous content in the pericardial space (arrow)

A 60-year-old male, with previous history of type 2 diabetes with mild renal insufficiency, presented to the emergency room (ER) with complaints of chest pain enhanced by respiratory motion for the past two days, with no fever or other symptoms. The electrocardiogram (ECG) showed diffuse and superiorly concave ST-segment elevation. His blood tests revealed an elevated C-reactive protein with no other significant findings and a transthoracic echocardiogram (TTE) was performed, showing a structurally normal heart, with preserved ejection fraction and a mild pericardial effusion. A diagnosis of uncomplicated pericarditis was made at this stage and the patient was discharged under colchicine 0.5 mg bid and acetylsalicylic acid 1,000 mg tid, with weaning off instructions, and a cardiology appointment was scheduled. However, the patient returned six days later, with persistency of intense chest and new onset of fever and fatigue. He was tachycardic and hypotensive. The ECG was similar to the previous one. The TTE showed a significant pericardial effusion, causing hemodynamic compromise. The patient was submitted to an emergent pericardiocentesis with hemodynamic stabilization. Blood cultures were drawn and the pericardial fluid was sent to cultural examination, revealing a leucocyte count of 152/µL. Empirical antibiotic therapy was initiated with vancomycin and ceftriaxone. Blood cultures came back negative, but a pathogenic agent was isolated in the pericardial fluid: *Dermabacter hominis*. As for the antimicrobial susceptibility test, there are no breakpoints to *D. hominis* and so the minimal inhibitory concentrations (MICs) were obtained and the breakpoints of Corynebacterium (EUCAST) were applied, given the resemblance of these agents. The same antibiotics were maintained, in accordance with the above mentioned susceptibility test. During the first 48h, the patient had a favorable evolution, however a re-elevation of C-reactive protein (CRP) occurred, along with reappearance of fever (>38°C) and chest pain. Heart computed tomography (CT) scan and transesophageal echocardiogram were unremarkable. Serial TTE assessments showed re-development of new pericardial effusion with non-pure fluid with content of heterogeneous appearance (Figure 1 (Fig. 1)). It is possible that the antibiotics used were not fully effective. Given the unfavorable evolution, the case was discussed in Heart Team and a surgical drainage with pericardiectomy was considered the best approach. The patient was submitted to a bilateral antephrenic pericardiectomy, with drainage of amorphous tissue from the pericardial sac. 

The patient had no recurrence of fever or chest pain, the inflammatory parameters decreased and there were no complications detected by echocardiography. There were no isolations of *Dermabacter hominis* in the blood cultures that followed. The patient was discharged after 42 days of antimicrobial therapy and no complications were detected at 1-year follow-up.

## Discussion

This is the first case report of bacterial pericarditis due to *Dermabacter hominis*. Not only is it an infrequent human infection agent, as it is usually associated with favorable clinical outcomes. Described as opportunistic, *D. hominis* is commonly related to immunocompromised patients [2]. Our case was very different: firstly, *D. hominis* was responsible for bacterial pericarditis, an aggressive and rare pathology, with its isolation in pericardial fluid, which had never been reported before; secondly, our patient was not particularly comorbid, having solely non-complicated diabetes; thirdly the response to dual antibiotic therapy was very poor with need for surgical intervention. Lastly, the patient’s blood cultures were negative. This raises the question of how *D. hominis* reached pericardial fluid. We raise the hypothesis that other sites in the human body may be sanctuaries for this agent. 

## Conclusion

The infections caused by this agent are uncommon. In current literature there are no reported associations of *Dermabacter hominis* and pericarditis or its isolation in pericardial fluid. The unfavorable evolution under dual antibiotic therapy, plus the need for surgical treatment, suggest it may be a particularly aggressive agent in the acute pericarditis setting. The absence of *D. hominis* in our patient’s blood cultures and its isolation in pericardial fluid raise the hypothesis that there may be sanctuaries in the human body. 

## Highlights

1. This is the first case in scientific literature reporting the documentation of *Dermabacter hominis* in pericardial fluid.
2. Given the unfavorable evolution and the need of a surgical approach, *Dermabacter hominis* seems to be an aggressive agent in the acute pericarditis setting, unlike what has been previously described in literature.
3. *D. hominis* is a skin colonizer. Given the exclusive isolation of *D. hominis* in pericardial fluid, with persistently negative blood cultures, we raise the hypothesis of existing sanctuary sites.

## Notes

### Acknowledgements

We thank the Department of Cardiology and the Department of Infectious Diseases of Hospital Professor Doutor Fernando Fonseca, Amadora, Lisbon, for their support in the management of the bacterial pericarditis case due to such an uncommon pathogenic agent. 

### Ethical approval statement

This case report conformed to the principles of the Helsinki Declaration. Ethical approval was obtained from the ethical committee of our institution.

### Competing interests

The authors declare that they have no competing interests.
